# The complete chloroplast genome of the *Amygdalus ferganensis* (Rosaceae) in Xinjiang, China

**DOI:** 10.1080/23802359.2019.1676670

**Published:** 2019-10-15

**Authors:** Zhongyu Du, Yizhong Duan

**Affiliations:** a Shaanxi Key Laboratory of Ecological Restoration in Northern Shaanxi Mining Area, Yulin University, Yulin, China;; b Breeding Base for State Key Laboratory of Land Degradation and Ecological Restoration in Northwest China, Ningxia University, Yinchuan, China;; c Ministry of Education Key Laboratory for Restoration and Reconstruction of Degraded Ecosystems in Northwest China, Ningxia University, Yinchuan, China;; d College of Chemistry and Materials Science, Northwest University, Xi’an, China

**Keywords:** *Amygdalus ferganensis*, complete chloroplast genome, phylogenetic tree

## Abstract

*Amygdalus ferganensis* is a member of the family Rosaceae. In this paper, we report complete chloroplast genome sequences of *A. ferganensis* (Rosaceae). The results showed that *A. ferganensis* complete chloroplast genome comprises 158,365 bp, containing a largen single copy (LSC) region of 86,240 bp, a small single copy (SSC) region of 19,012 bp, and a pair of inverted repeats (IRs) region of 26,386 bp. The genome has a GC content of 42.6%. The LSC, SSC, and IR regions represent 54.46, 12.01, and 33.32% of the *A. ferganensis* chloroplast genome length. We annotated 112 genes, including 78 protein coding genes, 4 rRNAs, and 30 tRNAs. And *A. ferganensis* is closely the *A. persica*.

*Amygdalus ferganensis* is widely distributed throughout Xinjiang, North, and South. In the southern Xinjiang, natural selection and artificial domestication in long-term; it has become one of the most characteristic fruit tree types in the region (Kelimu et al. 2017). As *A. ferganensis* is an important germplasm resource, many scholars have studied about morphology, cytology, palynology and enzymology, but we are not clear about the positioning of the classification of *A. ferganensis* still (Chen et al. 2015).

Chloroplast (cp) genome is one of the three sets of genetic systems (cytoblast, chloroplast, and mitochondrion) with different evolutionary histories and origins in higher plants, the chloroplast genomes have independent evolutionary routes and own the characteristics of uniparental inheritance, moderate rates of nucleotide substitutions, haploid status, and no homologous recombination (Hansen et al. 2007; Shaw et al. 2005). However, there is less research on the chloroplast genome characteristics of *A. ferganensis*; and, in this paper, we report complete chloroplast genome sequences of *A. ferganensis* (Rosaceae). We hope to provide a theoretical basis for the chloroplast genome characteristics and the phylogenetic relationship of this species.

The fresh leaves of *A. ferganensis* were provided from Zhengzhou fruit research institute, CAAS (Chinese Academy of Agricultural Sciences) in Henan, China (113°38′E, 34°45′N) in June 2018. The specimens of *A. ferganensis* (Accession Number: 20180910Yl03) were deposited at the Herbarium of Yulin University, Shaanxi, China. Genomic DNA was extracted from the fresh leaves according to a modified CTAB method (Doyle and Doyle 1987), and the high-throughput sequencing was carried out using the Illumia HiSeq X Ten system. The complete chloroplast genome of *A. pedunculata* (MG869261) was used as the reference sequence to assembled and annotated. The complete chloroplast genome of *A. ferganensis* was annotated using the Geneious 8.0.2 (Kearse et al. 2012). The physical map of *A. ferganensis* was visualised using OGDRAW online tool (Lohse et al. 2013). The complete chloroplast genome sequences were aligned using MAFFT (Kazutaka et al. 2002), and we used the MEGA v7.0 (Kumar et al. 2016) to construct a phylogenetic tree according to the neighbor-joining method, with a bootstrap value of 1000. The complete chloroplast genome sequence of *A. ferganensis* has been deposited into the GenBank (MK798146).

Our result showed that complete chloroplast genome of *A. ferganensis* comprises 158,365 bp, containing a large single copy (LSC) region of 86,240 bp, a small single copy (SSC) region of 19,012 bp, and a pair of inverted repeats (IRs) region of 26,386 bp. The genome has a GC content of 42.6%. The LSC, SSC, and IR regions represent 54.46, 12.01, and 33.32% of the *A. ferganensis* chloroplast genome length. We annotated 112 genes, including 78 protein-coding genes, 4 rRNAs, and 30 tRNAs.

The 17 species of complete chloroplast genomes was constructed using a phylogenetic tree, and the *Platanus occidentalis* (DQ923116) complete chloroplast genome as an outgroup (Figure 1). The result showed that *A. ferganensis* is closely the *A. persica*.

**Figure 1.**
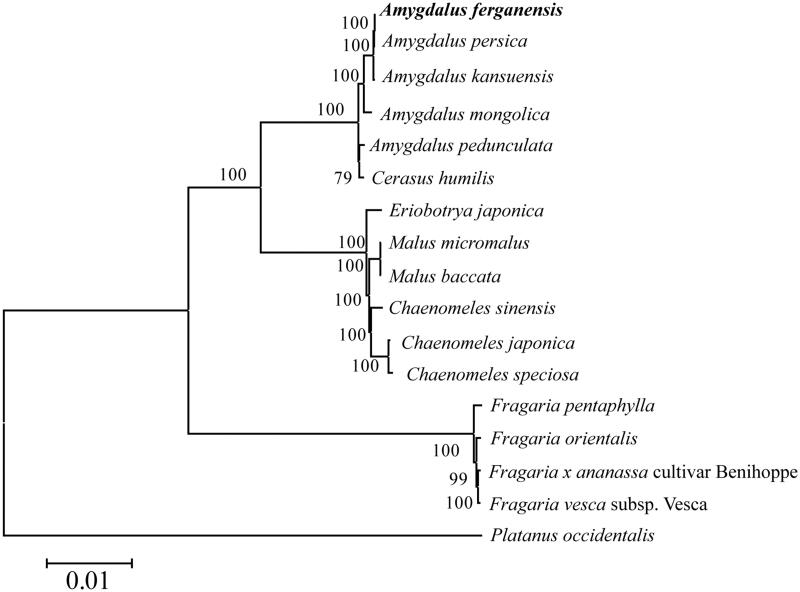
Phylogenetic tree constructed based on 17 species of chloroplast genomes. *Amygdalus mongolica* (KY073235); *Amygdalus pedunculata* (MG869261); *Amygdalus persica* (HQ336405); *Amygdalus kansuensis* (NC023956); *Amygdalus ferganensis* (MK798146); *Cerasus humilis* (NC035880); *Malus baccata* (KX499859); *Malus × micromalus* (NC036368); *Chaenomeles japonica* (NC035566); *Chaenomeles sinensis* (KT932967); *Chaenomeles speciosa* (KT932965); *Eriobotrya japonica* (NC034639); *Fragaria x ananassa* cultivar Benihoppe (KY358226); *Fragaria vesca* subsp. Vesca (JF345175); *Fragaria orientalis* (NC035501); *Fragaria pentaphylla* (NC034347); *Platanus occidentalis* (DQ923116).

## References

[CIT0001] Chen QJ, Meng D, Li W, Gu ZY, Duan XW, Yuan H, Zhang Y, Li TZ 2015 Cloning and analysis of S-RNase and SFBgenes in Xinjiang peach (*Prunus ferganensis* Kost.et Riab). Jou of Chi Agr Uni. 20(6):76–83. (In Chinese.)

[CIT0002] Doyle JJ, Doyle JL 1987 A rapid DNA isolation procedure for small quantities of fresh leaf tissue. Phyt Bul. 19:11–15.

[CIT0003] Hansen DR, Dastidar SG, Cai Z, Penaflor C, Kuehl JV, Boore JL, Jansen RK 2007 Phylogenetic and evolutionary implications of complete chloroplast genome sequences of four early-diverging angiosperms: Buxus (Buxaceae), Chloranthus (Chloranthaceae), Dioscorea (Dioscoreaceae), and Illicium (Schisandraceae). Mol Phy and Evo. 45(2):547–563. 10.1016/j.ympev.2007.06.004 17644003

[CIT0004] Kazutaka K, Kazuharu M, Kei-Ichi K, Takashi M 2002 MAFFT: a novel method for rapid multiple sequence alignment based on fast Fourier transform. Nucleic Acids Res. 30:3059–3066. 12136088 10.1093/nar/gkf436 PMC135756

[CIT0005] Kearse M, Moir R, Wilson A, Stones-Havas S, Cheung M, Sturrock S, Buxton S, Cooper A, Markowitz S, Duran C, et al. 2012 Geneious Basic: an integrated and extendable desktop software platform for the organization and analysis of sequence data. Bioinformatics. 28(12):1647–1649. 22543367 10.1093/bioinformatics/bts199 PMC3371832

[CIT0006] Kelimu YM, Han LQ, Maerhaba WSM, Aisikaer AHMT, Ghunqam ABDRXT, Abulimiti MS, Wang XW, Ma K, Wang JX 2017 Investigation of the fruit characters and preliminary evaluation of different Xinjiang peach germplasms. Xinjiang Agr Sci. 54(6):1041–1046. (In Chinese.)

[CIT0007] Kumar S, Stecher G, Tamura K 2016 Mega7: molecular evolutionary genetics analysis version 7.0 for bigger datasets. Mol Biol Evol. 33(7):1870–1874. 27004904 10.1093/molbev/msw054 PMC8210823

[CIT0008] Lohse M, Drechsel O, Kahlau S, Bock R 2013 Organellar Genome DRAW – a suite of tools for generating physical maps of plastid and mitochondrial genomes and visualizing expression data sets. Nucleic Acids Res. 41(W1):W575–W581. 23609545 10.1093/nar/gkt289 PMC3692101

[CIT0009] Shaw J, Lickey EB, Beck JT, Farmer SB, Liu W, Miller J, Siripun KC, Winder CT, Schilling EE, Small RL 2005 The tortoise and the hare II: relative utility of 21 noncoding chloroplast DNA sequences for phylogenetic analysis. Am J Bot. 92(1):142–166. 21652394 10.3732/ajb.92.1.142

